# Picking Apart a Program Evaluation Committee: A Multiple Case Study Characterizing Primary Care Residency Program Evaluation Committee Structure, Program Improvement, and Outcomes

**DOI:** 10.7759/cureus.57439

**Published:** 2024-04-02

**Authors:** Lacy E Lowry, Jerusalem Merkebu, Sarah E Schall, Brian E Neubauer, Alexis Battista

**Affiliations:** 1 Internal Medicine, San Antonio Uniformed Services Health Education Consortium (SAUSHEC), Fort Sam Houston, USA; 2 Health Professions Education, Henry M. Jackson Foundation for the Advancement of Military Medicine, Uniformed Services University of the Health Sciences, Bethesda, USA

**Keywords:** primary care, systems theory, graduate medical education, process improvement, program evaluation

## Abstract

Background: As of 2014, the Accreditation Council for Graduate Medical Education (ACGME) mandates initiating a Program Evaluation Committee (PEC) to guide ongoing program improvement. However, little guidance nor published reports exist about how individual PECs have undertaken this mandate.

Objective: To explore how four primary care residency PECs configure their committees, review program goals and undertake program evaluation and improvement.

Methods: We conducted a multiple case study between December 2022 and April 2023 of four purposively selected primary care residencies (e.g., family medicine, pediatrics, internal medicine). Data sources included semi-structured interviews with four PEC members per program and diverse program artifacts. Using a constructivist approach, we utilized qualitative coding to analyze participant interviews and content analysis for program artifacts. We then used coded transcripts and artifacts to construct logic models for each program guided by a systems theory lens.

Results: Programs adapt their PEC structure, execution, and outcomes to meet short- and long-term needs based on organizational and program-unique factors such as size and local practices. They relied on multiple data sources and sought diverse stakeholder participation to complete program evaluation and improvement. Identified deficiencies were often categorized as internal versus external to delineate PEC responsibility, boundaries, and feasibility of interventions.

Conclusion: The broad guidance provided by the ACGME for PEC configuration allows programs to adapt the committee based on individual needs. However, further instruction on program evaluation and organizational change principles would augment existing PEC efforts.

## Introduction

Effective program evaluation (PE) and subsequent process improvement (PI) interventions rely on robust methodologies, quality data, and capable individuals [1]. PE uses systematic methods to evaluate a program with the goal of program and process improvement [2]. In 2014, the Accreditation Council for Graduate Medical Education (ACGME) mandated that residency Program Directors (PDs) appoint a Program Evaluation Committee (PEC) to guide ongoing program evaluation and improvement. These efforts include evaluating a program's outcomes, strengths, challenges, and opportunities to facilitate strategic planning and generating the Annual Program Evaluation (APE) [3]. PECs ideally utilize outcome parameters (e.g., board scores, rotation feedback) and other data (e.g., informal feedback) to systematically assess the program’s quality and progress toward its goals and aims with the objective of continuous program improvement.

From the outset, the ACGME provided foundational operational functions of the PEC, including a minimal membership requirement of at least two program faculty members, one of whom must be core, and at least one resident member, as well as meeting frequency of at least once per year [3]; however, guidance for how committees should be structured and operated is minimal, often focusing on procedural activities. For example, "best PEC practices," such as PEC responsibilities and recommendations for optimizing the APE, were emphasized in both a handbook developed by Jordan et al. and a separate workshop on MedEdPORTAL [4,5]. In addition, the literature has limited data on individual PEC structure and function. Of the few published reports available, diverse methodologies are described, including retrospective comparisons of meeting minutes with APE outcomes, developing individualized goals based upon previous APE findings, and structured faculty and trainee feedback of rotation curricula [6-8]. Trombetta et al. described the PEC process for the Adult Cardiothoracic Anesthesiology Fellowship programs at Cleveland Clinic and the University of Pennsylvania; however, there was little elaboration on why the structure was chosen [9]. Given this lack of clarity, questions remain about how residency programs have implemented their PECs, how they are structured and operated, and what activities and resources are required to achieve their goals.

Another challenge of implementing PECs is the variety of potential PI methodologies in the literature and limited training in PE. For example, some of healthcare’s most cited PI methods include the Plan-Do-Study-Act (PDSA) cycle, Six Sigma, and Lean models [10]. Although these approaches structure the steps of process improvement, a systematic review by Taylor et al. found that many PDSA method studies failed to align with critical features (e.g., application of sequential, iterative cycles) [11]. Furthermore, medical educators frequently encounter competing responsibilities, limiting available time to dedicate to systematic PE, and may feel they need more training in PE approaches [12]. One surgical program noted the temptation to run on “autopilot” given the time and resource commitment required for program administration [13]. However, effective PE requires the implementation of PI principles [1].

It has been a decade since the ACGME mandated the implementation of PECs, but there remains a paucity of data on individual program execution. Examining how programs have implemented them could provide invaluable guidance to ACGME and the training programs they support. This study aimed to describe how four purposively selected military PECs were configured, how they operated and reviewed program goals, and if/how they structured PE and subsequent PI.

Theoretical frameworks

We approached this project from a constructivist stance (which maintains that knowledge is actively constructed rather than passively taken in) because we acknowledged that each independent program would be constructed and function in ways consistent with its local context. Furthermore, we believed that to create independent case descriptions, we too needed to approach our analysis and data collection from a constructivist approach. We selected two theoretical frameworks to guide our research: systems theory and logic modeling.

We selected systems theory, a non-linear framework emphasizing that the parts of a system are best understood when viewed holistically. Specifically, the entity being studied (in our case, the PEC) should be examined within the context of its relationship to those around it, including the processes occurring at the (micro) individual, (meso) group, and (macro) organizational levels [14,15]. For example, PECs involve micro-level (e.g., committee members), meso-level (e.g., utilization of group feedback), and macro-level (e.g., larger institutional factors) components that inform their overall configuration and task execution.

Logic models are used in PE to focus the analysis and description of a program’s theory of action (i.e., the assumptions about its capability and function) and make it visible. They also acknowledge programs’ systems characteristics, including their "inputs, activities, outputs and outcomes” and the dynamic interrelationships of these components [16]. Understanding and mapping these characteristics through systems theory and logic modeling, respectively, helped us address our primary purposes of elucidating how these PECs were configured, how they operated, and how these activities and resources enabled them to achieve subsequent program revisions.

## Materials and methods

Design

We conducted a multiple case study using Merriam’s approach to examine four primary care residency programs (internal medicine, family medicine, and pediatrics) across the United States military [17]. We opted to first focus on primary care programs given their distinctive set of training priorities when compared to surgical programs (non-procedural vs. procedural). Merriam’s case study approach emphasizes “an intensive, holistic description and analysis of a bounded phenomenon" [17,18]. Distinguishing characteristics of this methodology include that it is particularistic (i.e., focuses on one idea or event such as a PEC), descriptive (i.e., results in a thick description of the phenomena), and heuristic (i.e., uncovers new meanings, extends understanding, and confirms established knowledge) [17,18]. A case study is ideal for this investigation because PECs are bound by place (e.g., specific residency), persons (e.g., chair, committee members), and goals (e.g., evaluation and program improvements), which allows for the generation of rich descriptions of each program to uncover new meanings and understandings of PEC structure and operations. We used purposive sampling to include a diverse representation of primary care specialties, military branches, and program sizes [19].

Invitations to participate were sent to the PDs of selected programs between November 2022 and March 2023. Subsequent invitations were sent to additional participants within each program based on guidance from the PD or the PEC chair. Interviews and artifact collection occurred between December 2022 and April 2023 with programs representing all three branches of military medicine (Air Force, Army, Navy) situated throughout the United States. Participation was voluntary, and verbal informed consent was obtained. No compensation was provided. None of the researchers were directly associated with the programs that agreed to participate.

Data collection and analysis

We collected and analyzed multiple forms of data for each case. We interviewed four active committee members, including the PD, the PEC chairperson, a faculty member-at-large, and a resident member-at-large. The principal investigator (L.L.) conducted interviews using a semi-structured interview guide developed and piloted by the research team. Questions focused on the structure, function, program improvement processes, program outcomes, and challenges; minor iterative question adjustments were made based on early interviews (see Appendices). Interviews were conducted virtually using Zoom (Zoom Video Communications Inc., San Jose, CA, USA) and ranged between 30-70 minutes. All interviews were audio recorded and then transcribed using Otter.ai (Otter.ai, Inc., Mountain View, CA, USA); following transcription, L.L. reviewed transcripts for accuracy. We also requested documents from each program. Programs determined which and how many documents to share, but we advised them to identify those that contained insights relevant to our research questions (e.g., program operations and goals). Each program shared four to 12 documents representing previous APEs, surveys, meeting agendas and minutes, and communication between members.

The principal investigator (L.L.) read and inductively coded all transcripts and artifacts after the data for each case was fully collected. The remaining team members (B.N., S.S., J.M, A.B.) were each assigned a case to review. Following individual coding, team members met every other week to share findings, discuss consistencies and differences in coding, and refine our coding. Drawing from the coded transcripts and program documents, we mapped codes onto each program’s logic model to compose descriptions of each case. Research team meetings were recorded and transcribed using Google Meet (Google LLC, Mountain View, CA, USA). Following these discussions, L.L. reviewed the transcripts, artifacts, and meeting transcripts and consolidated the identified codes into a final summary. We relied on logic modeling and systems theory to focus our analysis and descriptions of the PECs.

Reflexivity, validity, and ethics

The research team consisted of three team members with experience in Graduate Medical Education, including a current internal medicine PD (B.N.), a current internal medicine Associate PD (APD) (L.L.), and an internal medicine faculty member with extensive experience in PI (S.S.). Two team members were educational psychologists with expertise in health professions education (A.B., J.M.), PE and mixed and multiple methods research (A.B.), and organizational psychology and systems theory (J.M.). This diversity of experiences informed the process and analysis of the study and helped mitigate bias. Validity was addressed with triangulation through multiple investigators, multiple data sources, and peer examination [17]. The anonymity of the individual programs and participants was intentionally maintained to encourage an open forum for discussion that would create an authentic, in-depth analysis of each case. As such, specialty and military branch affiliations are not reported, and program sizes are given as ranges. The Uniformed Services University of the Health Sciences Institutional Review Board deemed this study exempt.

## Results

PEC configurations

PEC configurations were diverse (Table 1). Program size ranged from small to large, and PEC membership ranged from seven to 26. All programs comprised a combination of program leadership and resident members; however, only two programs (cases two and four) included members from other sub-specialties. PEC members were assigned based on interest or as a part of their role (e.g., APDs often were automatically assigned), while resident members were either self-nominated or were recommended by senior residents (e.g., chief residents).

**Table 1:** Summary of PEC configurations APD: Associate Program Director, PD: Program Director, PEC: Program Evaluation Committee, PGY: Postgraduate Year Program specialty is omitted to provide anonymity to participating programs. Program size reflects how each program describes the size of its program.

	Case 1	Case 2	Case 3	Case 4
Program Size	Small (8-12 Residents/PGY)	Small (4-7 Residents/PGY)	Medium (12-16 Residents/PGY)	Large (13-16 Residents/PGY)
Number of Members	7	10	19	26
PEC Composition	Program Director (PD), two APDs), two Chief Residents, and two residents (PGY-2 and PGY-3)	One APD, three faculty members from various subspecialties, and six residents (two per PGY); peripheral participation of a second APD and one Chief Resident	Seven program leadership (PD, APDs, chief residents), nine faculty, three resident members (one per PGY and one alternate)	Four program leadership (PD, three APDs), seven faculty members from various subspecialties, and fifteen resident members (seven PGY-2s, seven PGY-3s, one PGY-1)
PEC Chair	One APD	One APD	One Core Faculty Member	One APD
Meeting Cadence	Annual meeting to complete the PEC report, numerous ad hoc meetings	Four planned meetings, ad hoc meetings	Quarterly	Monthly for one hour and quarterly for three hours

There was considerable variation in meeting cadence, ranging from annually to monthly. Three of four programs reported meeting quarterly (cases two, three, and four). Programs one and two (smaller programs) supplemented formal meetings with ad-hoc meetings, which they reported as an ideal strategy for their smaller program size. These programs indicated that ad hoc meetings enabled them to be nimbler in addressing program issues.

PEC operations

Case one PEC convened face-to-face or via email annually to complete the APE as described by the PEC chair: “We don't really have a formal PEC throughout the year… we don't meet consistently.… [It's] really, once we get the ACGME survey, we all sit down together. So, it's busy for those couple of weeks to months, where we talk, at least, weekly about it, and we're putting the document together, but throughout the rest of the year, it's really, I would say, it's really informal.”

APDs and chief residents assumed membership based on their leadership roles, while resident members self-nominated following an email call to all program members; resident PEC members also served on the House Staff Council. The program had recently undergone leadership changes, with the PD in their first year and the APD/PEC chair in their second year.

Ongoing program improvement activities were identified and addressed via micro (e.g., one-on-one interactions between PEC members) and meso-level collaborations (e.g., resident PEC members held a 30-minute monthly meeting to discuss education issues such as rotations, faculty issues, and research opportunities). After monthly meetings, a memorandum for record (MFR) of the issues was drafted by one of the resident PEC members and discussed with the PD, with prioritization of these issues described as follows: “So, I prioritize them based on how important they are or how much I think I can, we can fix them. So, an example of one that I put at the very bottom is the fact that our cafeteria is only open, like Monday through Friday, for lunch, and not every other Friday and not holidays, and we have no other access to food, but, like, that's never going to change.”

**Figure 1:**
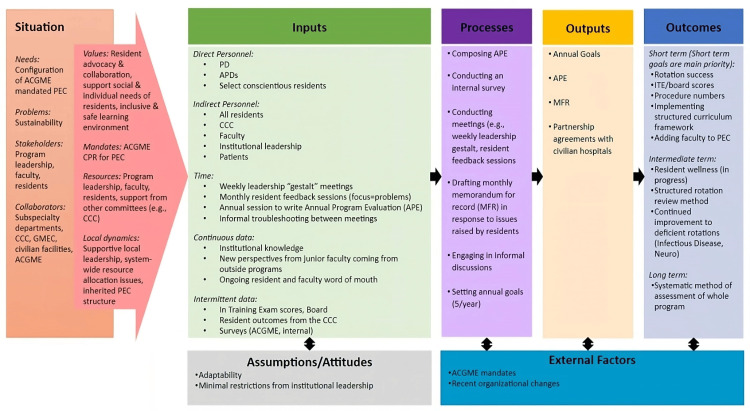
Case One Logic Model ACGME: Accreditation Council for Graduate Medical Education, APD: Associate Program Director, APE: Annual Program Evaluation, CCC: Clinical Competency Committee, CPR: Common Program Requirements, GMEC: Graduate Medical Education Committee, ITE: In-Training Exam, MFR: Memorandum for Record, PD: Program Director, PEC: Program Evaluation Committee

The PD, APDs (including the PEC chair), chief residents, and resident members met weekly to discuss program issues, including reviewing previously generated MFRs. During weekly meetings, issues were divided among the attendees to identify solutions and then shared with the PD. The PD then provided formal feedback to the residents and elevated issues to the GME committee when external stakeholder involvement was needed (e.g., hiring a transfer coordinator, and hospital climate issues such as cafeteria access). Select PEC-related items were “outsourced” to non-PEC faculty members (e.g., a faculty member acting as research advisor and leading an overhaul of the resident evaluation process). Of note, the PD was the only interviewee who identified these weekly meetings and outsourced activities as related to the PEC, while the other interviewees considered only the annual meeting to draft the APE. In addition to these activities, improvement needs were derived from direct engagement with the residents (particularly by chief residents) and individual resident assessments during Clinical Competency Committee meetings. For example, the program used curricular gaps identified by PEC using board and In-Training Exam (ITE) scores and resident feedback to establish new clinical partnerships to address these gaps (Figure 1).

Case two PEC had four scheduled meetings per academic year. Ad hoc meetings typically occur toward the year-end. The first meeting occurred during the year's first half to introduce the PEC’s role in rotation evaluation. The remaining meetings were held during the year's second half and focused on discussions of newly identified curriculum, learning environment issues (e.g., obtaining necessary procedural opportunities, academic session formatting), and updates on previously identified issues. The year's final meeting focused on a Strengths, Weaknesses, Opportunities, and Threats analysis supporting APE completion. The PD maintained a peripheral involvement in meetings to allow unconstrained member participation. This choice for this structure was described by the PD as follows: “I think you can do as little as meet once a year to just do an overview of your curriculum and that would probably meet the requirements, but we've tried to certainly engage and make it a little more formal so that we have a group of faculty that are very invested in program improvement and we each want to capitalize on that.”

**Figure 2:**
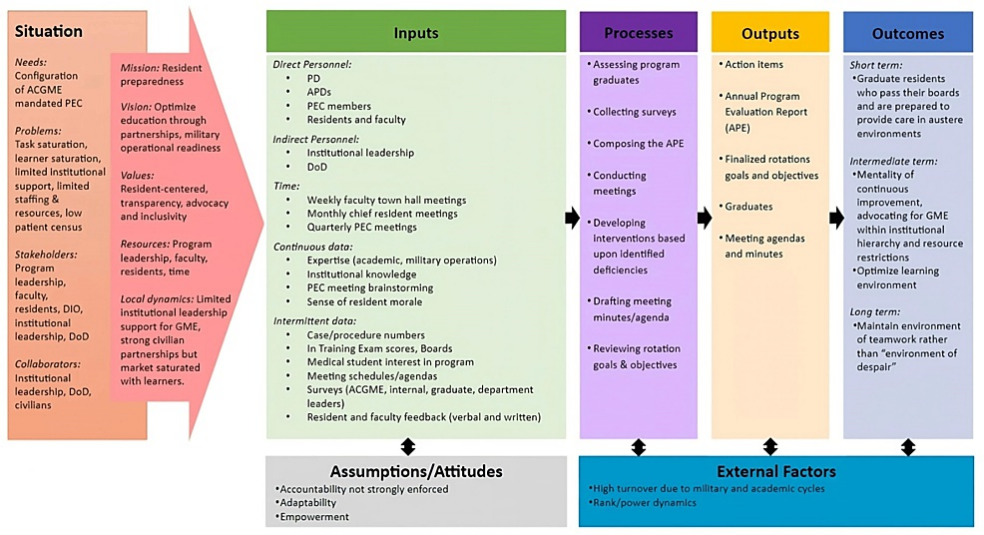
Case Two Logic Model ACGME: Accreditation Council for Graduate Medical Education, APD: Associate Program Director, APE: Annual Program Evaluation, CPR: Common Program Requirements, DIO: Designated Institutional Official, DoD: Department of Defense, GME: Graduate Medical Education, PD: Program Director, PEC: Program Evaluation Committee

The PD assigned faculty members based on interest in GME. Resident members self-nominated or were invited by the chief resident. Regarding roles and responsibilities in conducting the committee’s work, most of these PEC delegated activities were led by the two APDs because they had protected time. The PEC chair drafted the APE and then shared it with the PD for comments and edits. Faculty members reviewed rotation goals and objectives annually based on their specialty/interests. Deficiencies were identified using various methods, including survey data, direct feedback from residents (often solicited by resident committee members or the chief resident), weekly faculty “Town Hall” meetings, and ITE/board scores. After rotation modifications, the PEC followed up with residents and faculty members to evaluate implementation through interpersonal interactions and any available data (e.g., procedure logs) (Figure 2), which the PEC chair described as follows: “When we make that kind of change, we're pretty diligent on checking back in with them as far as how's the supervision? Are you-do you feel like you're being supported there? Do you feel like your schedules are given to you far enough in advance? Are you getting some meaningful feedback from them? So, whenever we make those kinds of changes, we do try to cycle right back to it.”

Case three had a committee focusing on policy and curriculum revisions, rotation deficiencies, and operational issues, with revisions decided by member vote before establishing the PEC. Following a major program leadership transition, including the PD and APDs, it initiated a PEC comprised of the policy and curriculum committee members. Whether the program intended to consolidate the two committees into one PEC or retain two separate entities was still under consideration. Resident members self-nominated to the initial policy and curriculum committee, while faculty membership origin was ambiguous.

At the time of the interview, the committee had met once, with representatives from each clinical section discussing areas for improvement (e.g., patient census/acuity, faculty feedback, standardization of faculty expectations for rotations), after which members discussed solutions and voted on final decisions. The PEC meeting was intentionally open to all program stakeholders; however, only committee members could participate in the final vote, described by a faculty-at-large member as follows: “I mean, they [program leadership] have to make a decision at the end of the day, but everyone votes. Everyone has their time to talk, to speak, and resident input is very important.”

The program was working toward a proactive approach to program improvement that reflected a bottom-up approach rather than one imposed by the administration, described by the PEC chair: “I think, with the PEC, this is more proactive, and this is more giving…instead of the exec committee coming down on us, it's more us bringing things more up to their level, and view so that they can see some of these things and go, 'I wasn't aware of that weakness', you know, or even-we are doing pretty good in this certain area.”

To achieve this, they employed multiple processes to gather member feedback, with the benefits of this highlighted by the PEC chair: “I do see it as a vehicle to really be able to, again, kind of be a process, because processes just allow things to not slip through the cracks. And to just make sure we keep moving on instead of cycling through the same things over and over because we forgot that we did them or just didn't do them, and now let's go back and, and try to bring that back to life again.”

**Figure 3:**
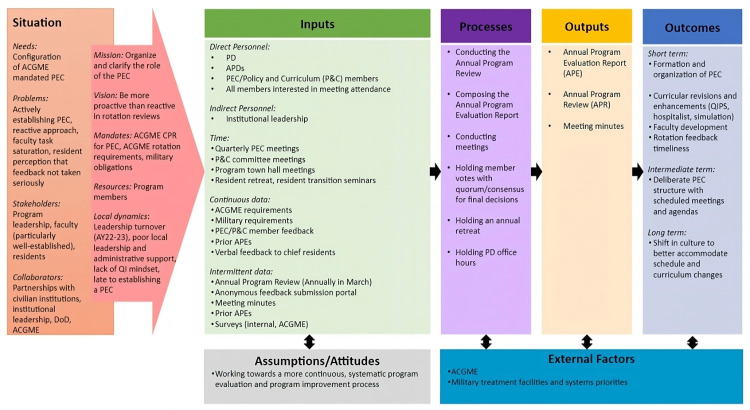
Case Three Logic Model ACGME: Accreditation Council for Graduate Medical Education, APD: Associate Program Director, APE: Annual Program Evaluation, APR: Annual Program Evaluation, CPR: Common Program Requirements, DoD: Department of Defense, P&C: Policy and Curriculum, PD: Program Director, PEC: Program Evaluation Committee, QI: Quality Improvement, QIPS: Quality Improvement and Patient Safety

Program deficiencies were identified via “informal” verbal feedback with chief residents, surveys (ACGME, internal), an annual resident retreat, an annual offsite program review, an anonymous feedback portal, and “Town Hall” meetings. The PD also recently initiated monthly “office hours” to encourage open discussion. Interventions (e.g., rotation modifications) were initiated by a final decision of the committee, and the formation of working groups (e.g., formalizing a parental leave policy). With the recent PEC structure transition, the PD planned to delegate sections to faculty members rather than complete the APE independently (Figure 3).

Case four viewed the PEC as a place for continuous growth and development and thus had robust processes to support their efforts. This focus on continuous growth was described by the PD: “…it’s a growing and learning organization. So, sometimes we try something, and it doesn't work. And so, we, you know, we change it. So, there's a lot there. And I think, you know, the other piece of the PEC is to take the experiences of the residents, the experience from the residents themselves, and address the gaps that they're experiencing.”

Their PEC met monthly for one hour and quarterly for three hours. Monthly meetings focused on rotation goals, learning objectives reviews, and finalizing decisions from prior discussions. Quarterly meetings addressed deeper issues, including major scheduling modifications or residency handbook development; the meetings were intentionally scheduled on Wednesdays when residents were present for didactics to encourage attendance. Meeting agendas were developed by the chair and provided to members in advance. The PD directed faculty membership based on desire and career interests, viewing PEC as a tool for faculty development. Resident members were nominated by classmates or self-nomination. The PD observed meetings, only providing input when prompted to encourage uninhibited participation.

The program coordinator drafted the APE, which was then revised by the PD and chair. Faculty members reviewed one to two rotation goals and objectives annually. These reviews were then submitted to the PEC chair for feedback before the meeting. Program deficiencies were identified using surveys (ACGME, graduate, internal), ITE/board scores, and verbal feedback, highlighted by a faculty-at-large member: "I mean, really, we just, we really ask our residents to give us, like, feedback constantly. We want to know, and as the postgraduate year (PGY)-1 APD, I make it very, very clear upfront that we value psychological safety, because we think that that's necessary for, you know, vulnerability, and growth, and trust…So, we as a group tend to embrace positive change, not change for sake of change, but, positive change that we talk it out and say this is why this change needs to happen.”

**Figure 4:**
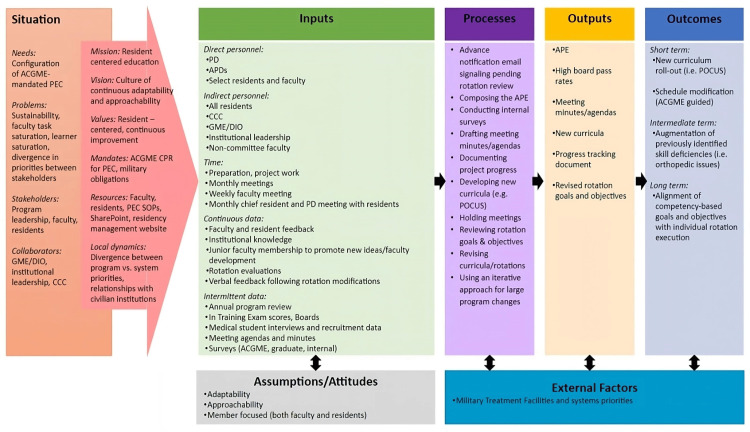
Case Four Logic Model ACGME: Accreditation Council for Graduate Medical Education, APD: Associate Program Director, APE: Annual Program Evaluation, CCC: Clinical Competency Committee, CPR: Common Program Requirements, DIO: Designated Institutional Official, GME: Graduate Medical Education, PD: Program Director, PEC: Program Evaluation Committee, POCUS: Point of Care Ultrasound, SOP: Standard Operating Procedures

Each spring, the program held a two-day Annual Program Review involving all faculty and residents. They completed a “magic wand” exercise using the theoretical idea of a limitless environment to elicit desired changes vs. preservation. Improvements were then prioritized based on feasibility and anticipated program impact. Various personnel, including the PD, PEC chair, and faculty members, undertook interventions based on their interests. Approximately two months after an intervention, residents' feedback was solicited to assess efficacy. The PEC chair maintained a color-coded document tracking the status of goals and objectives reviewed and completed interventions (Figure 4).

## Discussion

Program configuration, operations, goals, and PI efforts were diverse. Programs integrate program-unique factors such as faculty availability, experience, and openness to change to adapt their PEC structure, execution, and outcomes. Internal and external factors influenced PEC structure and function, including program size, leadership approaches, and institutional support. Such factors required navigating micro-, meso-, and macro-systems factors to achieve intervention success. PEC and program leadership recognize the value of proactive PE and program improvement, although some programs have limited support at the institutional level. PECs actively sought diverse stakeholder participation, including representation from a variety of residents, faculty, and others. These four programs used various data sources and methods, including multiple input activities, data from other committees, member redundancy, and analysis and decision-making methods. However, all programs desired enhanced guidance for foundational PEC structure and expressed that they lacked the formal knowledge, access to tools, and efficacy necessary to structure an effective PE and undertake the subsequent steps toward PI and organizational transformation.

PEC and program leadership recognized the importance of proactive PE and program change and expressed willingness to adapt. However, they often expressed lacking the formal knowledge, tools, and resources to undertake comprehensive PE and enact the subsequent transformation [13,20,21]. Nevertheless, they framed their efforts as iterative, continuously learning how to perform PE and PI using a deliberate, systematic approach.

Additionally, GME programs function within larger healthcare systems, adding further complexity [22]. Three cases (programs 1, 2, and 4) noted a tendency to prioritize micro (individual) and meso level (i.e., program) changes over macro (institutional) ones due to the increased difficulty and need for involvement of external stakeholders and increased bureaucracy. The findings are consistent with the literature highlighting the complex relationships in teaching hospitals [23]. Given the dynamic nature and high degree of scrutiny of medicine, organizational change requires the involvement of stakeholders within GME and the broader healthcare systems and a greater understanding of the system as a whole [23].

PE and improvement success depends on a holistic assessment of the dynamic interactions among micro, meso, and macro level processes to optimize intended outcomes while minimizing unintended negative consequences. Our case analysis approach to PEC shows the micro day-to-day process and macro level configurations that are essential for promoting ongoing improvements to each program. Mintrop et al. (2018) also support that a complex systems lens allows evaluations to “pick up on nuances that other evaluation approaches may overlook” [24]. Accordingly, our systems approach to PE generates and captures the dynamic and varied program-specific components and processes that must be taken into keen consideration for programmatic improvement [24]. Holistic stances counter prevailing reductionist approaches to change that involve dividing complex problems into component parts and are a foundational construct missing in the ACGME competency language [22,25,26]. Mizikazi suggests that when conducting PE, higher education should be viewed as a system with unique inputs, processes, and outputs [14]. Furthermore, goal setting and planning should consider the interactions between a PE and the system to maximize success and quality. Building upon the culture of adaptability (identified in all of these PECs), programs can be optimized by implementing a systems-oriented approach to PE.

One of the main limitations of this study is the focus on military primary care. Had we selected non-primary care or civilian programs, the findings may have differed. However, the findings help establish a foundation from which other PECs may reflect on their practices. Additionally, while military programs possess unique characteristics (e.g., dual role as military GME), all residency programs follow the same ACGME requirements; thus, we believe these findings can be useful to all programs. Finally, as in all qualitative research, prior experiences can influence data interpretation. We took numerous steps to mitigate bias by including multiple coders with diverse backgrounds, engaging in regular peer discussions, memoing, and recording all discussions. Future research should focus on non-primary care and civilian programs to augment the literature on PEC practices.

## Conclusions

Heightened visibility in individual Program Evaluation Committee (PEC) structures and knowledge of systematic approaches to program evaluation (PE) and organizational change can help solidify PEC best practices, enhancing existing PEC structures, which may translate to improved program quality, educational outcomes, and patient care. Our study showed that while programs endeavor to perform high-quality PE and process improvement, additional resources to support these efforts would be beneficial. Based on our findings, we recommend the following considerations. 

In terms of macro applications, programs should consider identifying resources to support junior faculty, such as access to established faculty development programs, developing an international medical education community of practice where tips and tricks can be shared among faculty of variable experience levels, or peer-to-peer observation and mentorship can be completed during the transition into new roles. In addition, the Accreditation Council for Graduate Medical Education and medical education community as a whole should consider enhancing existing program evaluation and improvement resources to assist busy and often inexperienced teaching faculty to perform expected tasks, including curriculum review and development, focused and global program assessments, and targeted interventions. In terms of meso applications, programs should focus on recognizing, developing, and maintaining relationships with all key stakeholders of their programs. In terms of micro applications, programs should consider identifying and supporting training and education courses in program evaluation, especially for PEC leadership. They should also encourage junior faculty participation in program evaluation and improvement efforts, using senior faculty members for initial and longitudinal guidance. 

## Appendices

Structured interview guide

*Background* 

The first few questions focus on your background to help better situate your experiences and responses.

1. Can you tell me about yourself, including your current role and educational and work experience leading up to your time serving on the Program Evaluation Committee (PEC)?  

2. If you have served in more than one PEC, please focus on your current PEC experience.    

3. How did you come to be a member of the PEC? 

4. How long have you been a PEC member?

5. What is the extent of your participation in this PEC to date?

The main interview questions then focus on five main areas: 

Structure and Function

How would you describe the structure of your PEC?

How many members does your PEC have? Why was that number was chosen? Do you think that this is an effective number of members? 

Who are the people and/or departments who make up your PEC?

Can you tell me about how these individuals and/or departments became involved in the PEC or PEC-related activities?

How active are your PEC members?

How active are these individuals?  (i.e. how often do they participate in these meetings, etc.) Can you give an example of these activities?

What kinds of resources and or supports (e.g., funding, technology) are available to the PEC you serve on?

From your perspective, what are the intended roles and responsibilities of the PEC?

How frequently does your PEC meet? What happens during your meetings?

How frequently does your PEC meet formally or informally?

What kinds of PEC activities happen between meetings (e.g., informal activities, follow up)?

What is your understanding of your role as an individual member of the PEC?  How does this compare to your colleagues’ (both faculty and trainees) role within PEC? 

Who do you think should serve on the PEC based upon your experience? Why?

For chair/Program Director (PD):

How do you recruit members? What are their expected roles as individuals? How long do members typically serve? Any particular challenges or best practices for this process?

Programmatic Improvement

Who contributes to Annual Program Evaluation (APE) completion?

How are areas of programmatic improvement identified, and can you share an example? How are they prioritized?

How does your PEC or program go about addressing identified areas for improvement?

Outcomes

How do you know if these interventions worked?

What steps are taken to evaluate changes after a program improvement effort is implemented?

What criteria does your PEC use to characterize a process improvement (PI) intervention as successful vs. unsuccessful vs. on-going?

If a change was considered to be unsuccessful, what happens next? 

How are programmatic improvements tracked over time?

How are changes communicated to residents and faculty?

What is the resident/faculty perception of programmatic improvement efforts?

Challenges

What kinds of challenges has your PEC experienced?

Have you noted any challenges that you feel may be unique to a military PEC (i.e. member deployments, etc.)?

Conclusion

Is there any additional information that I might have missed regarding your PEC experiences that might be helpful for me to know?
